# Thoracic radiotherapy (TRT) improved survival in both oligo- and polymetastatic extensive stage small cell lung cancer

**DOI:** 10.1038/s41598-017-09775-0

**Published:** 2017-08-23

**Authors:** Li-Ming Xu, Chingyun Cheng, Minglei Kang, Jing Luo, Lin-Lin Gong, Qing-Song Pang, Jun Wang, Zhi-Yong Yuan, Lu-Jun Zhao, Ping Wang

**Affiliations:** 1 Departments of Radiation Oncology, Tianjin Medical University Cancer Institute and Hospital, National Clinical Research Center for Cancer; Key Laboratory of Cancer Prevention and Therapy,Tianjin; Tianjin’s Clinical Research Center for Cancer, 300060 Tianjin, China; 2 Department of Radiation Oncology, University of Pennsylvania, Philadelphia PA, 19104 USA; 3 Department of Radiation Oncology, MedStar Georgetown University Hospital, 20007 Washington, DC, USA

## Abstract

There has been no previous study on the efficacy of the thoracic radiotherapy (TRT) in oligometastatic or polymetastatic extensive stage small-cell lung cancer (ES-SCLC) to the overall survival (OS). In a group of 270 ES-SCLC cases retrospective study, 78 patients (28.9%) had oligometastases and 192 (71.1%) had polymetastases, among which 51 oligometastatic patients (65.4%) and 93 polymetastatic patients (51.6%) received TRT. Propensity score matching (PSM) was utilized. The 2-year OS, progression free survival (PFS) and local control (LC) in oligometastatic and polymetastatic patients were 22.8% and 4.5% (p < 0.001), 12.0% and 3.8% (p < 0.001), and 36.7% and 6.1% (p < 0.001), respectively. The 2-year OS in oligometastatic patients with the chemotherapy + radiotherapy and chemotherapy alone were 25.2% and 12.7% (p = 0.002), in contrast to 10.0% and 6.8% (p = 0.030) in polymetastatic patients. The estimated hazard ratios for survival were 2.9 and 1.7 for both oligometastatic and polymetastatic patients with radiotherapy. The polymetastatic group has a lower LC (6.1% v.s. 36.7%, (p < 0.001)), due to polymetastases patients receiving involved-sites radiotherapy with low dose schemas. TRT improved OS of patients with oligometastases and polymetastases. Our study demonstrated that aggressive TRT might be a suitable addition of chemotherapy when treating ES-SCLC patients with oligometastases and polymetastases.

## Introduction

Cancer has been the leading cause of death and major public health problems in China as the morbidity and mortality rate increases, according to the Chinese cancer statistics in 2015^1^. Small cell lung cancer (SCLC) accounts for 12–15% of the total lung cancer cases^2, 3^. Approximately 60–70% of newly diagnosed SCLC cases are in extensive stage^1, 3^, and the prognosis of OS for ES-SCLC is poor. Some recent studies suggested that thoracic radiotherapy (TRT) play an important role in improving the survival in ES-SCLC^4–11^. However these studies only used chemotherapy response or extensive stage as the inclusion criteria.

Hellman *et al*.^12^ in 1995 first proposed the theory of oligometastases as a transitional state between localized and widespread systemic disease, while the number and site of metastatic tumors are limited. The clinical implication of this hypothesis is that localized forms of cancer treatment may be effective in patients with oligometastases. It has been affirmed in some literatures that oligometastases could be cured by local treatment such as colorectal cancer^13–15^, and studies have also confirmed that the local treatment could improve patients’ survival in NSCLC metastasis^16–18^. It has been reported that polymetastatic sites is an adverse prognostic factor for ES-SCLC^19^. To the best of our knowledge, there has no previous study about the role of TRT in ES-SCLC with oligometastases and polymetastases. In this study, we retrospectively analyzed the data from single-center to determine whether TRT could improve the prognosis of ES-SCLC patients with oligometastases or polymetastases.

## Methods

### Study patients and clinical data collection

This study was approved by the Regional Ethics Committee of Tianjin Medical University Cancer Institute & Hospital (NO. bc2017010). We confirm that all methods were carried out in accordance with relevant guidelines and regulations. This study is a retrospective analysis, and 270 patients from May 2010 to May 2015 with no previous ES-SCLC treatment were systemically reviewed. All the pathological types were demonstrated by morphological and immunohistochemical methods to conform with the neuroendocrine tumours of the lung diagnostic criteria^20^, and combined with clinical manifestations. Inclusion criteria include: (1) histopathological examination and immunohistochemistry were confirmed to be SCLC; (2) the state was stage IV (T any, N any and M1a/b); (3) patients did not receive prior treatment.

Staging criteria were according to American Joint Committee of Cancer (AJCC) 7^th^ edition manual. The pre-treatment staging examination included: complete medical history and physical examination, full blood count, serum biochemistry, chest X-ray, the computed tomography (CT) scans of the neck, chest, abdomen and pelvis, emission computed tomography (ECT)/positron emission tomography CT (PET-CT) scans and brain magnetic resonance imaging (MRI).

### The definition of oligometastases and polymetastases

The concept of oligometastases was first proposed in Hellman’s paper^12^, we further specified the ES-SCLC oligometastases as follows: (1) only one organ metastasis or metastatic lymph node metastases (able to be covered by a safe radiotherapy portal); (2) multiple brain metastases (treated with whole brain radiotherapy); or (3) continuous vertebral bone metastases treated in a single radiotherapy field. The ES-SCLC polymetastases were defined as the metastases excluding oligometastases.

### Treatment

ES-SCLC patients underwent platinum-contained chemotherapy with or without radiotherapy. The chemotherapy regimen was EP or CE regimen [cisplatin (DDP, 40 mg d1-3) or carboplatin (CBP, 400 or 500 mg d1) + etoposide (VP-16, 100 mg d1-5)]. The median number of chemotherapy cycles in all patients were 6 cycles.

For total 270 patients reviewed, 169 (62.6%) patients were in the chemotherapy responsive group, and 101 (37.4%) were in the non-chemotherapy responsive group. A total of 144 patients (53.7%) received radiotherapy after chemotherapy. Among these, 15 (10.4%) patients underwent the conservative anterior-posterior field radiotherapy, while 129 (89.6%) patients received intensity-modulated radiotherapy (IMRT) or 3D conformal radiotherapy (3DCRT). The conservative radiotherapy fields covered primary lesions, hilar and bilateral mediastinal lymph nodes. The gross tumour volume (GTV) included the primary lesion and positive metastatic lymph nodes. The clinical target volume (CTV) mainly included 0.5–0.8 cm of the primary lesion and the draining area of positive lymph nodes. The planned target volume (PTV) was defined as the CTV plus a 0.5–1.0 cm margin and PGTV (for all polymetastatic patients) was expanded from GTV with a 0.5–1.0 cm margin. The median dose was 45 Gy with the dose range of 30–60 Gy, and the fractionated radiotherapy dose was 1.8–3 Gy.

### Evaluation of the response to therapy

The response to the therapy was assessed following the response evaluation criteria in solid tumors^21^. The evaluation was performed every other cycle during the chemotherapy or 3 months after radiotherapy. The entire group of patients also received evaluation every 6–8 weeks post-treatment until disease progression.

### Follow-up and statistical analysis

The endpoints of the study included OS, PFS and LC, which were measured from the date of diagnosis till the date of events or last date of follow-up. The comparison of survival curves between the different groups was examined using the log-rank method, while the comparison of categorical data was performed by chi-square method. Multivariate analysis was performed using a Cox proportional hazard model. Because of the nonrandomized nature of this study, the data were matched and analyzed using PSM performed by SPSS 18.0 (SPSS Inc, Chicago, IL) software and R version 2.8.0 statistical package, for the control of confounding variables. P < 0.05 was considered to indicate a statistical significant difference.

## Results

### Patient characteristics

The clinical features of 270 patients were shown in Table 1. The ratio of male to female patients was 3.5:1.0, and the median age was 59 years (ranging from 18–85 years). 251 patients (93.0%) presented with good Karnofsky Performance Status score (KPS ≥80). 78 patients (28.9%) had oligometastases while 192 (71.1%) had polymetastases. 51 oligometastatic patients (65.4%) and 93 polymetastatic patients (51.6%) received TRT.

**Table 1:** Patient and disease characteristics of 270 patients.

Characteristic	No.	%
Age (years)		
<65 years	193	71.5
≥65 years	77	28.5
Sex, male	210	77.8
Smoking index, ≥400	214	79.3
Family history of neoplasm	54	20.0
Weight loss, >5%	25	9.3
KPS score, ≥80	251	93.0
T stage		
1	18	6.7
2	200	74.1
3	39	14.4
4	13	4.8
N stage		
0	3	1.1
1	20	7.4
2	157	58.1
3	90	33.3
Location of metastatic organs		
Brains	49	18.1
Others	221	81.9
PCI		
Yes	17	6.3
No	253	93.7
No. of ChT cycles		
1–3	35	13.0
≥4	235	87.0
Response to ChT		
Yes	169	62.6
No	101	37.4
TRT		
Yes	144	53.3
No	126	46.7

### Response to therapy and survival for all patients with oligometastases and polymetastases

The rates of complete response (CR), partial response (PR), stable disease (SD) and progression rate after the first course chemotherapy were 0 (n = 0), 62.6% (n = 169), 16.7% (n = 45) and 20.7% (n = 56), respectively. The rates after TRT are 0 (n = 0), 74.4% (n = 201), 5.6% (n = 15) and 20% (n = 54) respectively. The consolidative TRT significantly enhanced PR and reduced SD, with a comparable progression rate.

The 2-year OS, PFS and LC for all groups were 14.5%, 7.6% and 23.6% respectively for a median follow-up time of 36.4 months.

### Evaluation of candidate prognostic factors

Each clinical characteristic was assessed for prognostic significance against OS using the Kaplan-Meier method and COX regression analysis to evaluate whether there was an important relationship with prognosis. Patients having female gender, smoking index <400, oligometastases, chemotherapy cycles ≥4, response to chemotherapy, TRT and prophylactic cranial irradiation(PCI), showed favorable expectation using the univariate analysis. Other clinical features, such as age, family history of malignant tumors, weight loss >5%, KPS, state of stages, brain metastases and metastatic organs did not suggest any significant relationship to OS using the univariate analysis. Patients having oligometastases, chemotherapy cycles ≥4, response to chemotherapy and TRT showed favorable expectation using the COX regression analysis.

### Superior outcome with oligometastases

Patients with oligometastases were compared with those with polymetastases. The oligometastases and polymetastases groups were made comparable after PSM (see Table 2).

**Table 2:** Disease characteristics of patients with oligometastases vs. polymetastases after Propensity Score Matching.

Characteristic	Before Matching			After Matching		
	Oligometastases (n = 78)	Polymetastases (n = 192)	P	Oligometastases (n = 78)	Polymetastases (n = 78)	P
Age (years) ≥65	20	57	0.504	20	23	0.591
Sex, male	63	147	0.451	63	65	0.676
Smoking index, ≥400	61	153	0.785	61	66	0.303
Family history of neoplasm	11	43	0.123	11	17	0.211
Weight loss, >5%,	8	17	0.719	8	8	1.000
KPS score, ≥80	72	179	0.788	72	75	0.303
No. of ChT cycles			0.722			0.632
1–3	11	24		11	9	
≥4	67	168		67	69	
Response to ChT			0.023			0.089
Yes	57	112		57	57	
No	21	80		21	21	
TRT			0.011			1.000
Yes	51	93		51	51	
No	27	99		27	27	
PCI			0.615			0.731
Yes	4	13		4	5	
No	74	179		74	75	

The difference in survival between the two groups was statistically significant. The 2-year OS, PFS and LC in patients with oligometastases and polymetastases were 22.8% and 4.5% (p < 0.001); 12.0% and 3.8% (p < 0.001); and 36.7% and 6.1% (p < 0.001), respectively.

### Superior outcome with TRT in oligometastases and polymetastases

The authors compared oligometastatic and polymetastatic patients with chemotherapy + TRT to those with only chemotherapy. The bias due to the cofounding variables between group of chemotherapy with TRT and chemotherapy alone were reduced after PSM (see Tables 3 and 4).

**Table 3:** Disease characteristics of patients with oligometastases with chemotherapy + thoracic radiotherapy vs. chemotherapy after Propensity Score Matching.

Characteristic	Before Matching			After Matching		
	ChT + TRT (n = 51)	ChT (n = 27)	P	ChT + TRT (n = 22)	ChT (n = 22)	P
Age (years) ≥65	37	26	0.011	7	4	0.488
Sex,male	36	22	0.295	20	21	0.635
Smoking index, ≥400	40	21	0.947	20	19	1.000
Family history of neoplasm	8	3	0.581	4	3	0.680
Weight loss, >5%,	8	0	0.030	5	0	0.018
KPS score, ≥80	47	25	0.945	20	20	1.000
No. of ChT cycles			0.029			0.680
1–3	4	7		4	3	
≥4	47	20		18	19	
Response to ChT			0.297			1.000
Yes	32	25		13	13	
No	9	12		9	9	
PCI			0.135			0.148
Yes	4	0		2	0	
No	47	27		20	22

**Table 4:** Disease characteristics of patients with polymetastases with chemotherapy + thoracic radiotherapy vs. chemotherapy after Propensity Score Matching.

Characteristic	Before Matching			After Matching		
	ChT + TRT (n = 93)	ChT (n = 99)	P	ChT + TRT (n = 73)	ChT (n = 73)	P
Age (years) ≥65	23	34	0.145	18	24	0.273
Sex,male	72	75	0.786	59	55	0.424
Smoking index, ≥400	69	84	0.067	57	60	0.534
Family history of neoplasm	19	24	0.527	16	16	1.000
Weight loss, >5%,	11	6	0.160	10	5	0.173
KPS score, ≥80	88	91	0.456	69	71	0.404
No. of ChT cycles			0.548			0.596
1–3	13	11		9	7	
≥4	80	88		64	66	
Response to ChT			0.023			0.505
Yes	62	50		34	25	
No	31	49		39	48	
PCI			0.033			0.085
Yes	10	3		7	2	
No	83	96		66	71	

**Figure 1:**
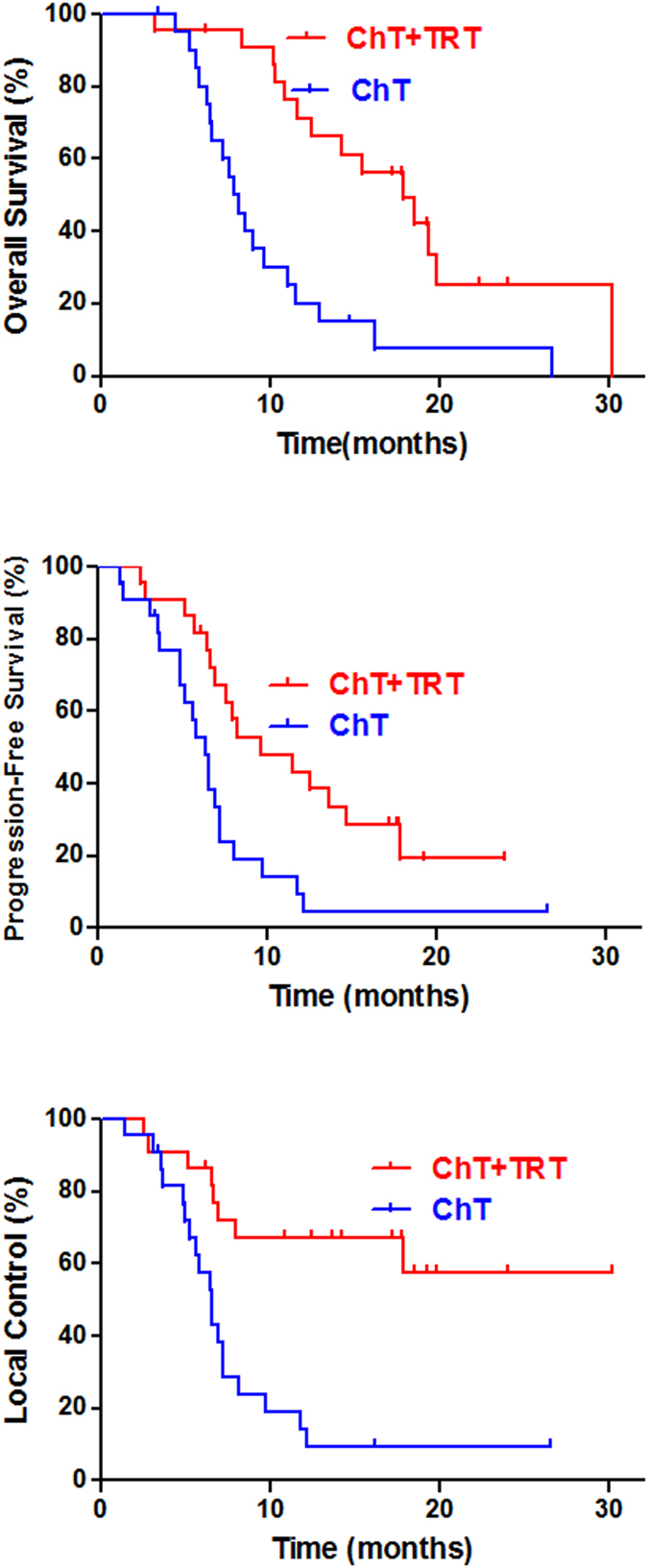
Thoracic radiotherapy improved the 2-year OS, PFS, and LC for oligometastatic SCLC patients.

The 2-year OS, PFS, and LC in oligometastatic patients treated with the chemotherapy + radiotherapy and the chemotherapy alone were 25.2% and 12.7% (p = 0.002); 19.3% and 4.8% (p = 0.006); and 57.6% and 9.6% (p < 0.001), respectively. The estimated hazard ratio for survival with chemotherapy + radiotherapy as compared with chemotherapy alone was 2.9 (95%CI, 1.4 to 6.0). Figure 1 shows the estimated survival distribution according to treatment group.

**Figure 2:**
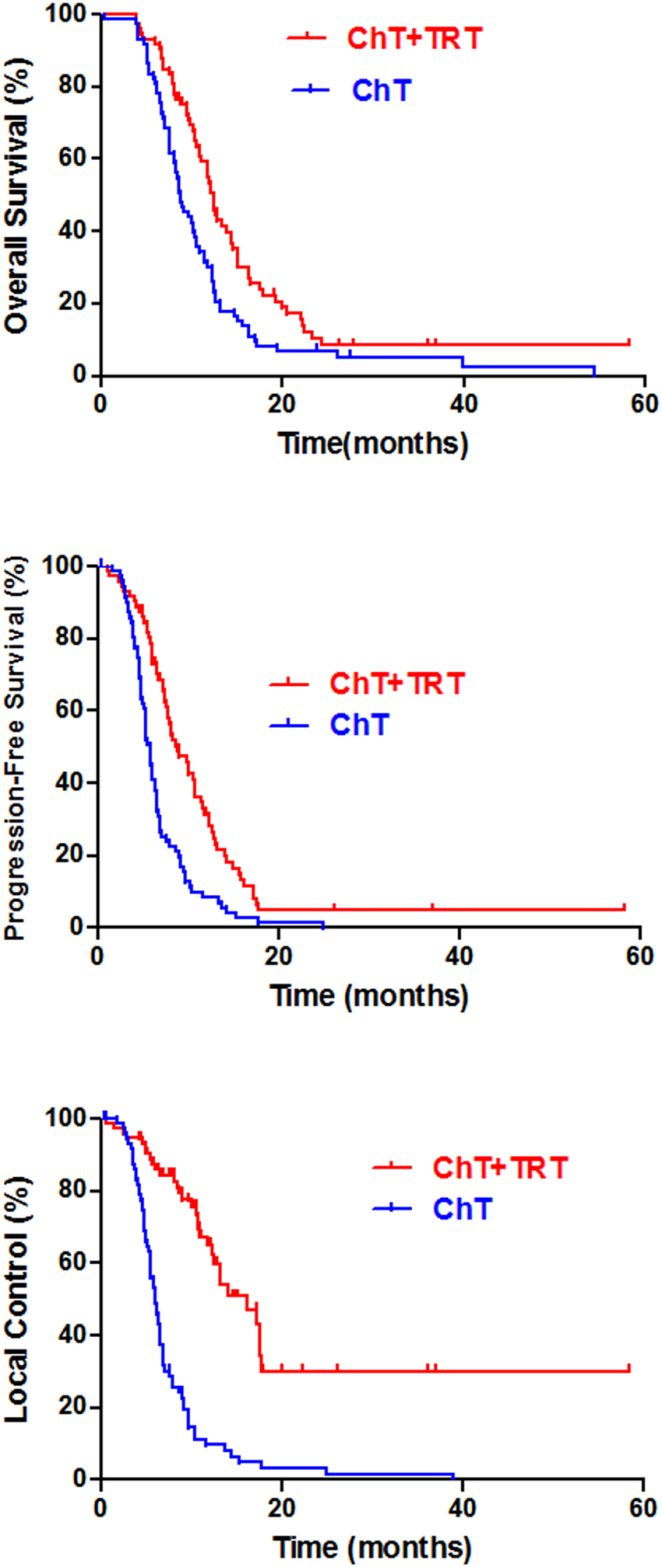
Thoracic radiotherapy improved the 2-year OS, PFS, and LC for polymetastatic SCLC patients.

The 2-year OS, PFS, and LC in polymetastatic patients treated with the chemotherapy + radiotherapy and chemotherapy alone were 10.0% and 6.8% (p = 0.030); 5.0% and 1.4% (p < 0.001); and 32.3% and 1.6% (p < 0.001), respectively. The estimated hazard ratio for survival with chemotherapy + radiotherapy as compared with only chemotherapy was 1.7 (95%CI, 1.2 to 2.3). Figure 2 shows the estimated survival distribution according to treatment group.

## Discussion

For ES-SCLC patients, chemotherapy remained the standard therapeutic modality, with a median survival of 7-12 months^3^. As of today, the first-line chemotherapy for ES-SCLC is the platinum-based combination regimen. The response rate with EP regimen was as high as 70–90%^22^. A large number of sensitive SCLC cells died after chemotherapy, and then the chemotherapy-resistance cells were promoted to proliferate increasingly. As a result, leading to relapse and metastasis, therefore patients have higher recurrence^23^. Platinum-based chemotherapy combined with TRT significantly improved OS and PFS in ES-SCLC patients^4, 16, 20, 24, 25^.

In the past when applying simple platinum-based chemotherapy as a standard treatment for ES-SCLC, thoracic radiotherapy was used only to palliate local symptoms and to improve quality of life^26^. Several clinical trials have demonstrated platinum-based chemotherapy combined with TRT to improve the prognosis of ES-SCLC patients^4, 16, 20, 24, 25^. According to the previous literatures^4, 16, 20, 24, 25^, the 2-year OS of patients with ES-SCLC who received chemotherapy plus TRT increased from 13% to 38%. For patients with a single chemotherapy regimen, when treated with additional TRT, the 2-year OS shows enhancement from 6% to 21%^4, 10, 16, 25^. It clearly shows that radiotherapy significantly improves the prognosis of patients with ES-SCLC, however the number of tumor metastasis were not considered in the studies.

Individualized treatment of NSCLC has been widely adopted, such as the targeted therapy, or radical treatment for patients with few oligometastases^27–29^. Resection or radiotherapy become a treatment option that provides high local control with minimal morbidity. De Ruysscher *et al*. reported their result of radical treatments (chemotherapyplus radiotherapy or surgery) for NSCLC patients with less than five oligometastases^30^, and the results indicating radical treatment had long-term PFS. Recently, Endo *et al*.^31^ reported a prospective phase II study of surgery for primary lesions (cT1-2N0-1) and surgery for synchronous or metachronous single organ metastases (e.g., brain, lung or others) with promising outcomes. Niibe *et al*.^32^ reviewed previous studies of oligometastatic NSCLC and advocated that local treatment approaches such as surgery or ablative radiation are only indicated for isolated brain or adrenal gland metastasis in terms of the survival results, and the most favorable lesion is 12 metachronous (recurrent) metastases. Punglia *et al*.^33^ demonstrated the hypothetical benefit of local therapy on the survival with increasing effectiveness of systemic therapy. Most reports concerning oligometastasectomy have showed that NSCLC with oligometastases can benefit from aggressive local therapy due to less biologically aggressive cancers. There was rare literature to report that the different role of TRT in oligometastatic and polymetastatic patients. Therefore, we analyzed the prognosis of ES-SCLC patients with oligometastases and polymetastases, and focused on whether TRT could improve the survival in ES-SCLC patients with different metastases. An interesting finding in this study is that the combination of platinum-based chemotherapy and TRT significantly improved the prognosis of ES-SCLC patients with oligometastases and polymetastases. Because of the high response rate (near 70%), the polymetastases maybe well treated. So TRT also significantly improves the prognosis of patients with polymetastases. The estimated hazard ratio for survival of oligometastatic patients with chemotherapy + radiotherapy was 2.9 compared with only chemotherapy; while that was 1.7 in polymetastatic patients. And the oligometastatic patients with chemotherapy + radiotherapy had superior survival benefits than polymetastatic patients. The 2-year OS in oligometastatic patients treated with the chemotherapy + radiotherapy and only chemotherapy were 25.2% and 12.7% (p = 0.002), and those were 10.0% and 6.8% (p = 0.030) in polymetastatic patients. In the meanwhile, the RTOG 0937 demonstrated a delay in progression of disease but no improve in 1-year OS with addition of consolidative extra-cranial irradiation^34^. Slotman *et al*.^35^ also reported that patients with 0–2 distant metastases, receiving TRT had significantly longer PFS (HR = 2.02; p = 0.003), but patients with more than 2 distant metastases, receiving TRT had not significantly longer PFS (HR1.25; p = 0.14); TRT did not lead to a significant benefit in OS in patients with 0–2 distant metastases or more than 2 distant metastases.

Another interesting finding of LC is that the actual LC in oligometastatic patients was superior to that in polymetastatic patients (36.7% v.s. 6.1%, (p < 0.001)), but the LC should be similar theoretically. We usually use involved-field radiotherapy (involved-sites radiotherapy usually in polymetastases) with low dose, so this was not improved in-field control and the LC was inferior in patients with polymetastases, and the local recurrences were still higher in patients with oligometastases. So we suggested that patients with oligometastases and polymetastases should receive the more aggressive TRT.

The limitations of this study include the small number of patients and retrospective analysis method. Therefore, some large prospective studies with dedicated designed chemotherapy and radiotherapy dose schemes are needed to confirm the findings. The optimal radiotherapy fields and prescription of ES-SCLC also need to be further studied.

## Conclusion

Overall, the combination of platinum-based chemotherapy and radiation therapy can improve OS and PFS in ES-SCLC patients with oligometastases or polymetastases. Patients with oligometastases compared to polymetastases benefited more from radiotherapy. The optimal options of thoracic radiotherapy in ES-SCLC need to be prospectively investigated to obtain more convincing evidence.
